# Supplementary material for: Glial Plasticity and Metabolic Stability After Knockdown of Astrocytic Cx43 in the Dorsal Vagal Complex
Source: Cells. 2025 Oct 29;14(21):1694. doi: 10.3390/cells14211694 (PMC12610448; doi:10.3390/cells14211694)
Supplement: Supplementary file 1 [file cells-14-01694-s001.zip › cells-3897309-supplementary.pdf]

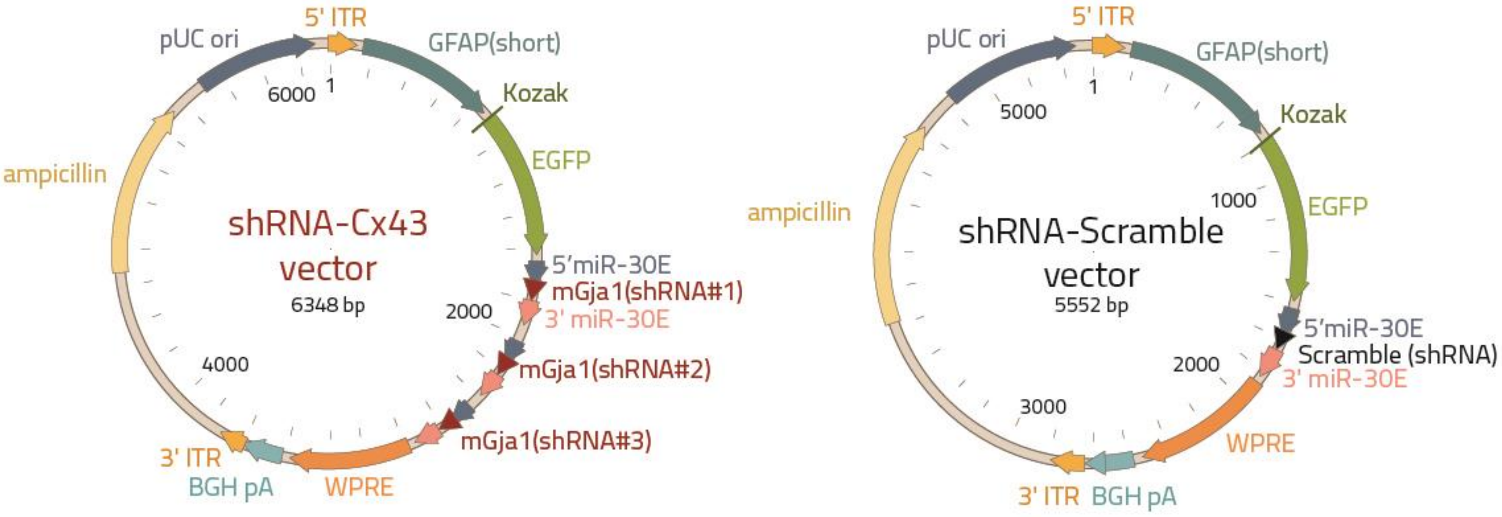

	**ShRNA-Cx43**	**ShRNA-Scramble**
Vector name	pAAV[3miR30]-<br>GFAP(short)>EGFP:{mGja1[shRN<br>A#1]}:{mGja1[shRNA#3]}:<br>{mGja1_shRNA}:WPRE	pAAV[miR30]-<br>GFAP(short)>EGFP:Scramble[miR30-<br>shRNA#1]:WPRE
Vector size	6348 bp	5552 bp
Target sequences by shRNA	GCCTGATGACCTGGAGATTTA<br>A<br>ACAATTCCTCCTGCCGCAATT<br>A<br>GAACAGTCTGCCTTTCGCTGT<br>A	ACCTAAGGTTAAGTCGCCCTCG

Figure S1: Composition of the plasmids used for generating the AAV-SHRNA-CX43 and AAV-SHRNA-SCRAMBLE vectors.

3' ITR: AAV 3' inverted terminal repeat for viral genome replication and recovery from the recombinant plasmid; **5' ITR**: AAV 5' inverted terminal repeat for viral genome replication and recovery from the recombinant plasmid; **5' miR-30E**: sequence facilitating the formation of mature shRNA and initiating gene knockdown; **Ampicillin**: ampicillin resistance gene allowing the selection of bacteria (*E. coli*) that have incorporated the vector; **BGH pA**: Bovine growth hormone polyadenylation signal, allowing transcription termination and polyadenylation of mRNA transcribed by RNA polymerase Pol II; **GFAP (short)**: Astrocyte-specific promoter; **EGFP**: Enhanced green fluorescent protein, optimized from a wild-type GFP variant of the jellyfish *Aequorea victoria*; **Kozak**: Kozak sequence for translation initiation; **pUC ori**: origin of replication for plasmid amplification in *E. coli*, ensuring high plasmid copy number; **WPRE**: Woodchuck hepatitis virus post-transcriptional regulatory element which improves viral stability transgene expression.

Gène	Primer Sequence		Target Site	qPCR T°	Amplicon Size	mRNA Identifier
**ywhaz**	F	ACATCTGCAACGATGTACTGTCT	680-702 (Exon 3-4)	60.0°C	166 bp	NM_001253805
	R	TGCTGTGACTGGTCCACAAT	826-845 (Exon 4)			
**mGjb2** (Cx26)	F	AGCCGTCTTCATGTACGTCTTT	697-718 (Exon 2)	60.0°C	124 bp	NM_008125.3
	R	CTTTTCTGTGGGCCTGGAAATG	799-820 (Exon 2)			
**mGjb6** (Cx30)	F	GGCCAACTGAGAAAACGGTG	1247-1266 (Exon 3)	60.0°C	56 bp	NM_008125.3<br>P000002.166.f ou r
	R	GCAAATCACGGATGCGGAAA	1283-1302 (Exon 3)			
**Gjb1 (cx32)**	F	GTGGACCTATGTCATCAGTGTGG	449-471 (exon 2)	60.0°C	119 bp	NM_001302496
	R	GGAAGGCTTCACACTTGACCAG	546-567 (exon 2)			
**Cx36**	F	TGATTGGGAGGATCCTGTTGAC	547-568 (exon 1)	60.0°C	95 bp	BC058595
	R	CATGGTCTGCTCATCATCGTAC	620-641 (exon 1)			
**mGja1** (Cx43)	F	GGTGGACTGCTTCCTCTCAC	817-836 (Exon 2)	60.0°C	151 bp	NM_010288.3
	R	ATCGCTTCTTCCCTTCACGC	948-967 (Exon 2)			
**Gjc1** (Cx45)	F	TTGGGTAACAGGAGTTCTGGTGA	789-812 (Exon 2)	60.0°C	145 bp	NM_008122.2
	R	GTGAGCCAGATCTTCCCTACA	892-912 (Exon 3)			
Gjc2 (Cx47)	F	GAGGATGAGGACGAGGAACCA	1275-1295 (exon 1)	60.0°C	111 bp	XM_036156249.1
	R	CACCGTCTTTCCATCACCTCC	1365-1385 (exon 1)			
**Panx1**	F	CAGGCTGCCTTTGTGGATTC	667-686 (Exon 2)	60.0°C	145 bp	NM_019482
	R	CGGGCAGGTACAGGAGTATG	792-811 (Exon 3)			
**GLT1 (Slc1a2)**	F	CCAACAATATGCCCAAGCAGG	616-636 (exon2)	60.0°C	155 bp	NM_001077514
	R	TGCTCCCAGGATGACACCAA	751-770 (exon 2-3)			
**GLAST (Slc1a3)**	F	CACTGCTGTCATTGTGGGTA	731-750 (exon 2-3)	60.0°C	125 bp	NM_ 600111
	R	CCATTCCTGTGACGAGACT	873-891 (exon 3-4)			
**mGluR1 (Grm1)**	F	AGGGCGATGCTTGATATCGT	1038-1057 (exon2)	60.0°C	88 bp	NM_001114333
	R	CCATTCCACTCTCGCCGTAA	1106-1125 (exon3)			
**mGluR2 (Grm2)**	F	GCGGCTCCTACAGTGATGTC	641-661 (exon 2)	60.0°C	134 bp	NM_001160353
	R	TCATAACGGGACTTGTCGCTC	756-775 (exon3)			
**mGluR3 (Grm3)**	F	ACCAAGCTCTGTGATGCAATG	2245-2265 (exon4)	60.0°C	142 bp	NM_001417964
	R	TCCCGTCTCCGTAAGTGTCA	2367-2386(exon5)			
**mGluR4 (Grm4)**	F	GAAGGTGCAGTCACCATTCTTC	1959-1980 (exon5)	60.0°C	101 bp	NM_001291045
	R	AACCAGATGTTGCGCCTGTT	2040-2059 (exon6)			
**mGluR5 (Grm5)**	F	CCCGAGCCATGGTAGACATA	1311-1330 (exon3)	60.0°C	140 bp	NM_001143834
	R	AGAGTGGGCGATGCAAATCC	1431-1450 (exon4)			
**mGluR6 (Grm6)**	F	CCTGCTGTTGGCACTGTGA	1752-1770 (exon 9)	60.0°C	114 bp	NM_173372
	R	GACGGCAGCCAGTGTGGTT	1752-1770 (exon 9)			
**mGluR7 (Grm7)**	F	GGCATTGGGCTGGAATTATGT	912-932 (exon 2)	60.0°C	127 bp	NM_177328
	R	AATTCTCACGGACTGGGCAAT	1018-1038 (exon 3)			
**mGluR8 (Grm8)**	F	TACAGCTCCTtGGAGTGTT	2546-2564 (exon 9)	60.0°C	71 bp	NM_001361125
	R	CTGTTCtCCATAGTCAATGAT	2596-2616 (exon 9)			

Table S1: Primers sequences used for SYBR Green assays.

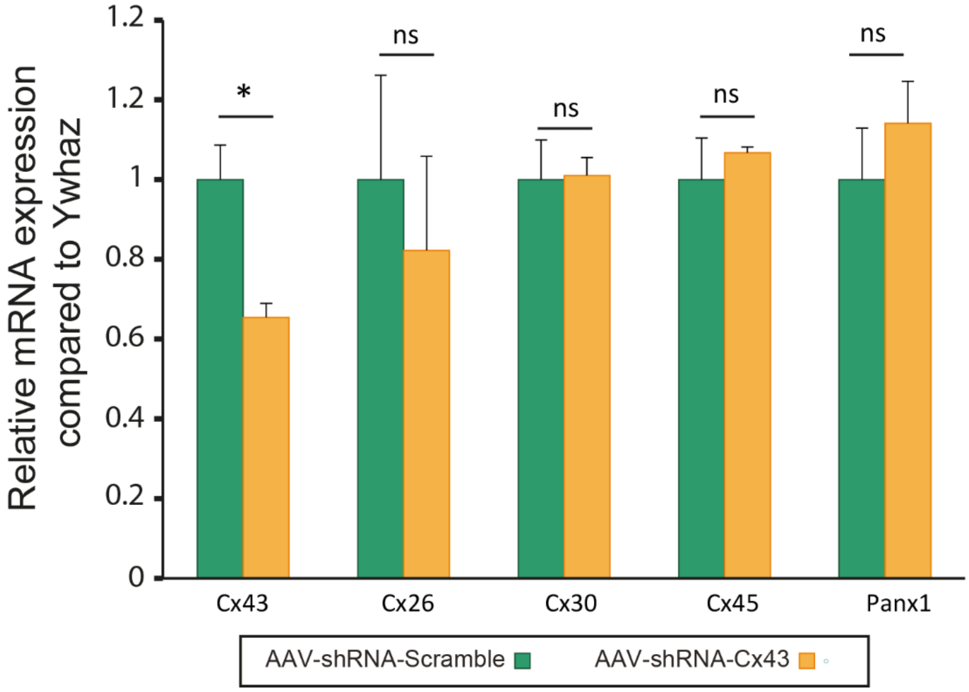

Figure S2: RT-qPCR quantification of connexins (Cx) and pannexin 1 (Panx1) expression levels in the DVC, normalized by YwHAz gene, at four weeks post-injection. Data are presented as mean ± SEM. AAV-shRNA-Scramble (n=6); AAV-shRNA-Cx43 (n=5). * $p<0.05$ indicate significant difference to control mice receiving AAV-shRNA-Scramble.
